# Supplementary material for: The Efficacy of Workplace Interventions on Improving the Dietary, Physical Activity and Sleep Behaviours of School and Childcare Staff: A Systematic Review
Source: Int J Environ Res Public Health. 2020 Jul 11;17(14):4998. doi: 10.3390/ijerph17144998 (PMC7400238; doi:10.3390/ijerph17144998)
Supplement: Supplementary file 1 [file ijerph-17-04998-s001.pdf]

Table S1. Search strategies per database

Database(s): **Ovid MEDLINE(R) and Epub Ahead of Print, In-Process & Other Non-Indexed Citations and Daily**
1946 to October 31, 2019

#	Searches
1	schools/ or schools, nursery/
2	Child Day Care Centers/
3	(pre-school* or preschool*).mp.
4	((childcare* or child care* or daycare* or day care*) adj (centre* or center* or worksite* or workplace* or work site* or work place*)).mp.
5	((high or middle or junior or primary or elementary or senior or prep*) adj school*).mp.
6	(school* adj3 (worksite* or workplace* or work site* or work place*)).tw,kw.
7	kindergarten*.tw,kw.
8	1 or 2 or 3 or 4 or 5 or 6 or 7
9	(teacher* or principal* or teach* assistant* or school director* or teach* aide*).mp.
10	((staff or employee* or worker* or workforce or personnel) adj3 (school* or preschool* or pre school* or kinder*)).mp.
11	9 or 10
12	sleep/
13	Sleep Deprivation/
14	Sleep*.ti,ab,kw.
15	exercise/ or gymnastics/ or physical conditioning, human/ or running/ or swimming/ or walking/
16	Motor Activity/
17	physical fitness/ or cardiorespiratory fitness/
18	life style/ or healthy lifestyle/ or healthy aging/ or healthy diet/ or sedentary lifestyle/
19	exp sports/
20	physical* inactiv*.ti,ab,kw.
21	physical* activ*.ti,ab,kw.
22	sedentary.ti,ab,kw.
23	Exercis*.ti,ab,kw.
24	Sport*.ti,ab,kw.
25	(("lifestyle*" or life style*) adj5 activ*).ti,ab,kw.
26	(fitness adj (class* or regime* or program*)).mp.
27	exp Diet/
28	nutrition*.ti,ab,kw.
29	Healthy eating.ti,ab,kw.
30	fruit*.ti,ab,kw.
31	Fruit/
32	Vegetable*.ti,ab,kw.

33	Vegetables/
34	Canteen*.ti,ab,kw.
35	Food services/ or food service*.ti,ab,kw.
36	Menu planning/ or menu*.ti,ab,kw.
37	calorie*.ti,ab,kw.
38	Energy intake/
39	energy density.ti,ab,kw.
40	eating/
41	Feeding Behavior/ or feeding behavio*.ti,ab,kw.
42	dietary intake.ti,ab,kw.
43	food habits.ti,ab,kw.
44	food/
45	Carbonated Beverages/ or soft drink*.ti,ab,kw.
46	soda.ti,ab,kw.
47	sweetened drink*.ti,ab,kw.
48	Dietary Fats, Unsaturated/ or Dietary Fats/
49	confectionar*.ti,ab,kw.
50	(school adj (lunch* or meal*)).ti,ab,kw.
51	((feeding or food or nutrition*) adj program*).ti,ab,kw.
52	cafeteria*.ti,ab,kw.
53	(food adj2 (purchas* or select*)).ti,ab,kw.
54	(well being or wellbeing).ti,ab,kw.
55	health promotion/ or health promotion.tw,kw.
56	12 or 13 or 14 or 15 or 16 or 17 or 18 or 19 or 20 or 21 or 22 or 23 or 24 or 25 or 26 or 27 or 28 or 29 or 30 or 31 or 32 or 33 or 34 or 35 or 36 or 37 or 38 or 39 or 40 or 41 or 42 or 43 or 44 or 45 or 46 or 47 or 48 or 49 or 50 or 51 or 52 or 53 or 54 or 55
57	8 and 11 and 56
58	animals/ not (humans/ and animals/)
59	57 not 58
60	limit 59 to english language
61	infection/ or apnea/ or allergy/ or surgery/
62	(infect* or allerg* or apnea* or apnoea* or surger*).tw,kw.
63	61 or 62
**64**	**60 not 63**

Database(s): **Embase**
1947 to October 31, 2019

#	Searches
1	school/ or high school/ or kindergarten/ or middle school/ or nursery school/ or primary school/
2	(pre-school* or preschool*).mp.
3	((childcare* or child care* or daycare* or day care*) adj (centre* or center* or worksite* or workplace* or work site* or work place*)).mp.
4	((high or middle or junior or primary or elementary or senior or prep*) adj school*).mp.
5	(school* adj3 (worksite* or workplace* or work site* or work place*)).tw,kw.
6	kindergarten*.tw,kw.
7	1 or 2 or 3 or 4 or 5 or 6
8	school teacher/
9	(teacher* or principal* or teach* assistant* or school director* or teach* aide*).mp.
10	((staff or employee* or worker* or workforce or personnel) adj3 (school* or preschool* or pre school* or kinder*)).mp.
11	8 or 9 or 10
12	sleep/ or sleep deprivation/
13	Sleep*.ti,ab,kw.
14	exercise/
15	gymnastics.mp. or physical education/
16	running/
17	swimming/
18	walking/
19	motor activity/
20	fitness/
21	cardiorespiratory fitness/
22	lifestyle/
23	healthy lifestyle/
24	healthy aging/
25	healthy diet/
26	sedentary lifestyle/
27	exp sport/
28	physical* inactiv*.ti,ab,kw.
29	physical* activ*.ti,ab,kw.
30	sedentary.ti,ab,kw.
31	Exercis*.ti,ab,kw.
32	Sport*.ti,ab,kw.
33	(("lifestyle*" or life style*) adj5 activ*).ti,ab,kw.

34	(fitness adj (class* or regime* or program*)).mp.
35	exp diet/
36	nutrition*.ti,ab,kw.
37	Healthy eating.ti,ab,kw.
38	fruit*.ti,ab,kw.
39	fruit/
40	Vegetable*.ti,ab,kw.
41	vegetable/
42	Canteen*.ti,ab,kw.
43	catering service/
44	food service*.ti,ab,kw.
45	menu*.ti,ab,kw.
46	calorie*.ti,ab,kw.
47	caloric intake/
48	energy density.ti,ab,kw.
49	eating/
50	feeding behavior/
51	feeding behavio*.ti,ab,kw.
52	dietary intake.ti,ab,kw.
53	food habits.ti,ab,kw.
54	food/
55	carbonated beverage/
56	soft drink*.ti,ab,kw.
57	soda.ti,ab,kw.
58	sweetened drink*.ti,ab,kw.
59	Dietary Fats.mp. or fat intake/
60	confectionar*.ti,ab,kw.
61	(school adj (lunch* or meal*)).ti,ab,kw.
62	((feeding or food or nutrition*) adj program*).ti,ab,kw.
63	cafeteria*.ti,ab,kw.
64	(food adj2 (purchas* or select*)).ti,ab,kw.
65	(well being or wellbeing).ti,ab,kw.
66	health promotion/
67	health promotion.tw,kw.
68	12 or 13 or 14 or 15 or 16 or 17 or 18 or 19 or 20 or 21 or 22 or 23 or 24 or 25 or 26 or 27 or 28 or 29 or 30 or 31 or 32 or 33 or 34 or 35 or 36 or 37 or 38 or 39 or 40 or 41 or 42 or 43 or 44 or 45 or 46 or 47 or 48 or 49 or 50 or 51 or 52 or 53 or 54 or 55 or 56 or 57 or 58 or 59 or 60 or 61 or 62 or 63 or 64 or 65 or 66 or 67
69	7 and 11 and 68

70	animal/ not (human/ and animal/)
71	69 not 70
72	limit 71 to english language
73	infection/ or apnea/ or allergy/ or surgery/
74	(infect* or allerg* or apnea* or apnoea* or surger*).tw,kw.
75	73 or 74
**76**	**72 not 75**

Database(s): **PsycINFO**
1806 to October Week 4 2019

#	Searches
1	JUNIOR HIGH SCHOOLS/ or HIGH SCHOOLS/ or MIDDLE SCHOOLS/ or NURSERY SCHOOLS/ or SCHOOLS/ or ELEMENTARY SCHOOLS/
2	Child Day Care/ or Nursery Schools/
3	(pre-school* or preschool*).tw,id.
4	((childcare* or child care* or daycare* or day care*) adj (centre* or center* or worksite* or workplace* or work site* or work place*)).tw,id.
5	((high or middle or junior or primary or elementary or senior or prep*) adj school*).tw,id.
6	(school* adj3 (worksite* or workplace* or work site* or work place*)).tw,id.
7	kindergarten*.tw,id.
8	1 or 2 or 3 or 4 or 5 or 6 or 7
9	PRESCHOOL TEACHERS/ or JUNIOR HIGH SCHOOL TEACHERS/ or TEACHERS/ or HIGH SCHOOL TEACHERS/ or STUDENT TEACHERS/ or ELEMENTARY SCHOOL TEACHERS/ or MIDDLE SCHOOL TEACHERS/
10	(teacher* or principal* or teach* assistant* or school director* or teach* aide*).tw,id.
11	((staff or employee* or worker* or workforce or personnel) adj3 (school* or preschool* or pre school* or kindergarten*)).tw,id.
12	9 or 10 or 11
13	SLEEP DEPRIVATION/ or SLEEP/
14	Sleep*.tw,id.
15	EXERCISE/
16	gymnastics.tw,id.
17	RUNNING/
18	SWIMMING/
19	WALKING/
20	Motor Activity.tw,id.
21	Physical Fitness/
22	cardiorespiratory fitness.tw,id.
23	exp Lifestyle/
24	healthy diet.tw,id.
25	healthy aging.mp.
26	exp SPORTS/
27	physical* inactiv*.tw,id.
28	physical* activ*.tw,id.
29	sedentary.tw,id.
30	Exercis*.tw,id.
31	Sport*.tw,id.

32	(("lifestyle*" or life style*) adj5 activ*).tw,id.
33	(fitness adj (class* or regime* or program*)).tw,id.
34	exp DIETS/
35	nutrition*.tw,id.
36	Healthy eating.tw,id.
37	fruit*.tw,id.
38	Vegetable*.tw,id.
39	Canteen*.tw,id.
40	food service*.tw,id.
41	menu*.tw,id.
42	calorie*.tw,id.
43	exp Food Intake/ or Energy intake.tw,id.
44	energy density.tw,id.
45	eating.tw,id.
46	feeding behavio*.tw,id.
47	dietary intake.tw,id.
48	food habits.tw,id.
49	FOOD/
50	soft drink*.tw,id.
51	Carbonated drink*.tw,id.
52	soda.tw,id.
53	sweetened drink*.tw,id.
54	Dietary Fats.tw,id.
55	confectionar*.tw,id.
56	(school adj (lunch* or meal*)).tw,id.
57	((feeding or food or nutrition*) adj program*).tw,id.
58	cafeteria*.tw,id.
59	(food adj2 (purchas* or select*)).tw,id.
60	(well being or wellbeing).tw,id.
61	health promotion.mp. or Health Promotion/
62	13 or 14 or 15 or 16 or 17 or 18 or 19 or 20 or 21 or 22 or 23 or 24 or 25 or 26 or 27 or 28 or 29 or 30 or 31 or 32 or 33 or 34 or 35 or 36 or 37 or 38 or 39 or 40 or 41 or 42 or 43 or 44 or 45 or 46 or 47 or 48 or 49 or 50 or 51 or 52 or 53 or 54 or 55 or 56 or 57 or 58 or 59 or 60 or 61
63	8 and 12 and 62
64	limit 63 to (human and english language)
65	(infect* or allerg* or apnea* or apnoea* or surger*).mp.
**66**	**64 not 65**

CINAHL

#	Query
S1	(MH "Schools") OR (MH "Schools, Middle") OR (MH "Schools, Secondary") OR (MH "Schools, Nursery") OR (MH "Schools, Elementary")
S2	(MH "Child Day Care") OR (MH "Child Care Providers")
S3	("pre-school*" or preschool*)
S4	((childcare* or "child care*" or daycare* or "day care*") n1 (centre* or center* or worksite* or workplace* or "work site*" or "work place*"))
S5	((high or middle or junior or primary or elementary or senior or prep*) n1 school*)
S6	(school* n3 (worksite* or workplace* or "work site*" or "work place*"))
S7	kindergarten*
S8	S1 OR S2 OR S3 OR S4 OR S5 OR S6 OR S7
S9	(teacher* or principal* or "teach* assistant*" or "school director*" or "teach* aide*")
S10	((staff or employee* or worker* or workforce or personnel) n3 (school* or preschool* or "pre school*" or kinder*))
S11	S9 OR S10
S12	(MH "Sleep")
S13	TI sleep* OR AB sleep*
S14	(MH "Exercise")
S15	(MH "Gymnastics")
S16	(MH "Running")
S17	(MH "Swimming")
S18	(MH "Walking")
S19	(MH "Motor Activity")
S20	(MH "Physical Fitness")
S21	(MH "Cardiorespiratory Fitness")
S22	(MH "Life Style") OR (MH "Life Style, Sedentary")
S23	("healthy ag?ing" or healthy diet")
S24	(MH "Sports+")
S25	("physical* inactiv*"or "physical* activ*")
S26	TI sedentary OR AB sedentary
S27	TI (Exercis* or sport*) OR AB (Exercis* or sport*)
S28	TI (((lifestyle* or "life style*") n5 activ*)) OR AB (((lifestyle* or "life style*") n5 activ*))
S29	(fitness n1 (class* or regime* or program*))
S30	(MH "Diet+")
S31	TI nutrition* OR AB nutrition*
S32	TI "Healthy eating" OR AB "Healthy eating"

S33	TI fruit* OR AB fruit*
S34	(MH "Fruit")
S35	TI Vegetable* OR AB Vegetable*
S36	TI Canteen* OR AB Canteen*
S37	(MH "Food Services")
S38	TI food service* OR AB Food service*
S39	(MH "Menu Planning")
S40	TI calorie* OR AB calorie*
S41	(MH "Energy Intake")
S42	(MH "Energy Density")
S43	(MH "Eating") OR (MH "Eating Behavior")
S44	TI feeding behavio* OR AB feeding behavio*
S45	TI dietary intake OR AB dietary intake
S46	(MH "Food Habits")
S47	(MH "Food")
S48	(MH "Carbonated Beverages")
S49	TI "soft drink*" OR AB "soft drink*"
S50	TI soda OR AU soda
S51	TI "sweetened drink*" OR AB "sweetened drink*"
S52	(MH "Dietary Fats")
S53	TI confectionar* OR AB confectionar*
S54	TI ((school n1 (lunch* or meal*))) OR AB ((school n1 (lunch* or meal*)))
S55	TI (((feeding or food or nutrition*) n1 program*)) OR AB (((feeding or food or nutrition*) n1 program*))
S56	TI cafeteria* OR AB cafeteria*
S57	TI ((food n2 (purchas* or select*))) OR AB ((food n2 (purchas* or select*)))
S58	TI (("well being" or wellbeing)) OR AB (("well being" or wellbeing))
S59	(MH "Health Promotion")
S60	TI "health promotion" OR AB "health promotion"
S61	S12 OR S13 OR S14 OR S15 OR S16 OR S17 OR S18 OR S19 OR S20 OR S21 OR S22 OR S23 OR S24 OR S25 OR S26 OR S27 OR S28 OR S29 OR S30 OR S31 OR S32 OR S33 OR S34 OR S35 OR S36 OR S37 OR S38 OR S39 OR S40 OR S41 OR S42 OR S43 OR S44 OR S45 OR S46 OR S47 OR S48 OR S49 OR S50 OR S51 OR S52 OR S53 OR S54 OR S55 OR S56 OR S57 OR S58 OR S59 OR S60
S62	S8 AND S11 AND S61
S63	(MH "Infection")
S64	(MH "Apnea")
S65	(MH "Allergy and Immunology")

S66	(MH "Hypersensitivity")
S67	(MH "Surgery, Operative")
S68	TI ((infect* or allerg* or hypersensitivit* or apnea* or apnoea* or surger*)) OR AB ((infect* or allerg* or hypersensitivit* or apnea* or apnoea* or surger*))
S69	S63 OR S64 OR S65 OR S66 OR S67 OR S68
**S70**	**S62 not S69 english and humans**

EBSCO MegaFile Ultimate – search in title and subject terms fields

Preschool* or "pre school*" or ((childcare* or "child care*" or daycare* or "day care*") n1 (centre* or center* or worksite* or workplace* or "work site*" or "work place*")) or ((high or middle or junior or primary or elementary or senior or prep*) n1 school*) or (school* n3 (worksite* or workplace* or work site* or work place*)) or kindergarten*<br><br>teacher* or principal* or "teach* assistant*" or "school director*" or "teach* aide*" or ((staff or employee* or worker* or workforce or personnel) n3 (school* or preschool* or "pre school*" or kinder*))<br><br>sleep* or exercis* or gymnastic* or running or swimming or walking or "motor activity" or "physical fitness" or "cardiorespiratory fitness" or "life style" or lifestyle or "healthy ag?ing" or "healthy diet" or sport* or "physical activit*" or "physical inactivit*" or sedentary or "fitness class*" or "fitness regime*" or "fitness program*" or diet or nutrition* or "healthy eating" or fruit* or vegetable* or canteen* or "food service*" or menu* or calorie* or "energy intake" or "energy density" or eating or "feeding behavio*" or "dietary intake" or food or "carbonated drink*" or "soft drink*" or soda or "sweetened drink*" or "dietary fat*" or confectionar* or "school lunch*" or "school meal*" or ((feeding or food or nutrition*) n1 program*) or cafeteria* or (food n2 (purchas* or select*)) or wellbeing or "well being" or "health promotion"

SportDiscus – search in title, abstract and subject fields

Preschool* or "pre school*" or ((childcare* or "child care*" or daycare* or "day care*") n1 (centre* or center* or worksite* or workplace* or "work site*" or "work place*")) or ((high or middle or junior or primary or elementary or senior or prep*) n1 school*) or (school* n3 (worksite* or workplace* or work site* or work place*)) or kindergarten*<br><br>teacher* or principal* or "teach* assistant*" or "school director*" or "teach* aide*" or ((staff or employee* or worker* or workforce or personnel) n3 (school* or preschool* or "pre school*" or kinder*))<br><br>sleep* or exercis* or gymnastic* or running or swimming or walking or "motor activity" or "physical fitness" or "cardiorespiratory fitness" or "life style" or lifestyle or "healthy ag?ing" or "healthy diet" or sport* or "physical activit*" or "physical inactivit*" or sedentary or "fitness class*" or "fitness regime*" or "fitness program*" or diet or nutrition* or "healthy eating" or fruit* or vegetable* or canteen* or "food service*" or menu* or calorie* or "energy intake" or "energy density" or eating or "feeding behavio*" or "dietary intake" or food or "carbonated drink*" or "soft drink*" or soda or "sweetened drink*" or "dietary fat*" or confectionar* or "school lunch*" or "school meal*" or ((feeding or food or nutrition*) n1 program*) or cafeteria* or (food n2 (purchas* or select*)) or wellbeing or "well being" or "health promotion"

Dissertations and Theses – anywhere except Fulltext

Preschool* or “pre school*” or ((childcare* or “child care*” or daycare* or “day care*”) near/1 (centre* or center* or worksite* or workplace* or “work site*” or “work place*”)) or ((high or middle or junior or primary or elementary or senior or prep*) near/1 school*) or (school* near/3 (worksite* or workplace* or work site* or work place*)) or kindergarten*

teacher* or principal* or “teach* assistant*” or “school director*” or “teach* aide*” or ((staff or employee* or worker* or workforce or personnel) near/3 (school* or preschool* or “pre school*” or kinder*))

sleep* or exercis* or gymnastic* or running or swimming or walking or “motor activity” or “physical fitness” or “cardiorespiratory fitness” or “life style” or lifestyle or “healthy ag?ing” or “healthy diet” or sport* or “physical activit*” or “physical inactivit*” or sedentary or “fitness class*” or “fitness regime*” or “fitness program*” or diet or nutrition* or “healthy eating” or fruit* or vegetable* or canteen* or “food service*” or menu* or calorie* or “energy intake” or “energy density” or eating or “feeding behavio*” or “dietary intake” or food or “carbonated drink*” or “soft drink*” or soda or “sweetened drink*” or “dietary fat*” or confectionar* or “school lunch*” or “school meal*” or ((feeding or food or nutrition*) near/1 program*) or cafeteria* or (food near/2 (purchas* or select*)) or wellbeing or “well being” or “health promotion”

ERIC

Preschool* or “pre school*” or ((childcare* or “child care*” or daycare* or “day care*”) near/1 (centre* or center* or worksite* or workplace* or “work site*” or “work place*”)) or ((high or middle or junior or primary or elementary or senior or prep*) near/1 school*) or (school* near/3 (worksite* or workplace* or “work site*” or “work place*”)) or kindergarten*

teacher* or principal* or “teach* assistant*” or “school director*” or “teach* aide*” or ((staff or employee* or worker* or workforce or personnel) near/3 (school* or preschool* or “pre school*” or kinder*))

sleep* or exercis* or gymnastic* or running or swimming or walking or “motor activity” or “physical fitness” or “cardiorespiratory fitness” or “life style” or lifestyle or “healthy ag?ing” or “healthy diet” or sport* or “physical activit*” or “physical inactivit*” or sedentary or “fitness class*” or “fitness regime*” or “fitness program*” or diet or nutrition* or “healthy eating” or fruit* or vegetable* or canteen* or “food service*” or menu* or calorie* or “energy intake” or “energy density” or eating or “feeding behavio*” or “dietary intake” or food or “carbonated drink*” or “soft drink*” or soda or “sweetened drink*” or “dietary fat*” or confectionar* or “school lunch*” or “school meal*” or ((feeding or food or nutrition*) near/1 program*) or cafeteria* or (food near/2 (purchas* or select*)) or wellbeing or “well being” or “health promotion”

Scopus

TITLE-KEY (preschool* OR "pre school*" OR ((childcare* OR "child care*" OR daycare* OR "day care*") AND (centre* OR center* OR worksite* OR workplace* OR "work site*" OR "work place*")) OR ((high OR middle OR junior OR primary OR elementary OR senior OR prep*) AND school*) OR (school* AND (worksite* OR workplace* OR work AND site* OR work AND place*)) OR kindergarten*) AND TITLE-KEY (teacher* OR principal* OR "teach* assistant*" OR "school director*" OR "teach* aide*" OR ((staff OR employee* OR worker* OR workforce OR personnel) W/3 (school* OR preschool* OR "pre school*" OR kinder*))) AND TITLE-KEY (sleep* OR exercis* OR gymnastic* OR running OR swimming OR walking OR "motor activity" OR "physical fitness" OR "cardiorespiratory fitness" OR "life style" OR lifestyle OR "healthy ag?ing" OR "healthy diet" OR sport* OR "physical activit*" OR "physical inactivit*" OR sedentary OR "fitness class*" OR "fitness

regime*" OR "fitness program*" OR diet OR nutrition* OR "healthy eating" OR fruit* OR vegetable* OR canteen* OR "food service*" OR menu* OR calorie* OR "energy intake" OR "energy density" OR eating OR "feeding behavio*" OR "dietary intake" OR food OR "carbonated drink*" OR "soft drink*" OR soda OR "sweetened drink*" OR "dietary fat*" OR confectionar* OR "school lunch*" OR "school meal*" OR ((feeding OR food OR nutrition*) W/1 program*) OR cafeteria* OR (food W/2 (purchas* OR select*)) OR wellbeing OR "well being" OR "health promotion") AND (LIMIT-TO (LANGUAGE , "English")) AND (LIMIT-TO (EXACTKEYWORD , "Human") OR LIMIT-TO (EXACTKEYWORD , "Humans"))

Table S2.
Overview of intervention characteristics

	Chen, 2009	Kupolati, 2019	O'Loughlin, 1996	Resnicow, 1998	Siegel, 2010	Wang, 2016
**Theoretical framework**	Health promoting schools framework	Social cognitive theory, Meaningful learning model	NR	The Gimme-5 curriculum (administered to both groups) was based on Social Cognitive Theory. No theoretical model was stated for the teacher wellness program (administered to the intervention group).	Social cognitive theory	Health promoting schools framework
**Materials**	NR	Nutrition education (NE) materials including a manual, picture book, learner's workbook (for students), and posters.	Educational materials were provided to support desired behaviour change.	The Johnson and Johnson Live for Life Program included promotional materials, printed educational materials, and a series of interactive health workshops.	Formation of worksite wellness committees, interschool competitions, newsletters, cooking classes, and walking clubs.	Educational resources, formation of school nutrition groups and leaders (facilitators), school nutrition group meetings, nutrition training for teachers, promotional resources, posters, peer support activities.
**Procedures**	NR	Teachers were trained to use the contextual NE materials in a 1-day workshop at the end of the 2014 school year,	Four screening stations were set up in the school gymnasium or conference room, including: (a) a	In year 1, teachers were offered 36 health workshops, addressing topics such as weight loss,	Intervention schools were asked to conduct brief written surveys of employees to identify the types of	The HPS School implemented a wide range of health promotion activities using the

followed by a re-orientation session for teachers to refresh their understanding on how to use the manual.<br><br>The developed NE materials were implemented in the allotted periods and time set out for different topics in the school curriculum, starting in February and ending in October 2015. In total, teachers taught five nutrition topics.<br><br>For Grade 6, nutrition topics were taught for 5 weeks in February and March in 32 classes and for 2 weeks in October in nine classes. For Grade 5, nutrition topics were taught for 2 weeks in July and August in six classes. The duration of each class was 30 minutes.<br><br>While the topics were being taught, we visited the school to supply foods and materials for class demonstrations.<br><br>Teachers received feedback (via telephone) as implementation progressed.	registration area where subjects signed a consent form and completed a self-administered questionnaire; (b) a diet, physical activity, and smoking history assessment station where subjects completed an interviewer-administered dietary assessment adapted from the ‘‘Rate Your Plate’’ program of the Pawtucket Heart Health Program, as well as questions on current smoking and exercise behaviour; (c) a blood pressure and blood cholesterol measurement station; and (d) a counselling station where the results were reviewed and interpreted by a physician or nurse.<br><br>Participants who needed lifestyle changes were encouraged to set personal behavior change objectives in one or two areas, although all smokers were strongly encouraged to quit. Participants were urged to select objectives that they themselves	blood pressure control, and stress management, each approximately 30 minutes in length.<br><br>In year 2, 18 workshops were offered. An exercise program was also offered at each school. In most schools, teachers requested walking or low impact aerobics programs after school.<br><br>In addition, during year 2, in weeks when participation in classes was expected to be low (e.g., between Thanksgiving and winter break), wellness counsellors conducted schoolwide campaigns instead of workshops; for example, they offered health risk appraisals at a centrally located station or conducted highly visible health activities during which health education materials were distributed.<br><br>Participants also received personalised feedback incorporating their baseline physiologic results, and they were offered	wellness activities that interested them and their preferred scheduling.<br><br>Each intervention school formed a worksite wellness committee of volunteers to develop and implement health promotion activities for its employees. These committees (3–10 people) met about every 2 months (3–9 meetings in academic year) and primarily comprised teachers, but also included administrative staff (principal, assistant principal), other academic staff (teacher aides), and support staff (office and food service personnel, custodians).<br><br>A school liaison (employed by the research study) attended committee meetings, recorded minutes, followed up on action items, and offered technical assistance to the committees in carrying out their activities.<br><br>Contingent on providing school-level data to the researchers, each intervention	HPS framework. The core component of the intervention in the HPS School was determined using the 2011 Chinese Residents Dietary Guidelines (National Health and Family Planning Commission of China, 2011), which includes the definition and importance of a balanced diet, the functions of nutrients, nutrient deficiencies and their effects, how to supplement necessary nutrients reasonably, good hygienic practices and food safety.

The principal of the treatment school ensured an enabling atmosphere for the implementation.	felt were achievable. Simple, graded steps to achieve these objectives were described by the screening counsellor.	incentives (e.g., T-shirts) for attending TeachWell classes and increasing exercise as well.	school was given a stipend of $3500 per year (for 3 years) to subsidize its wellness activities. Each control school was given an unrestricted stipend of $1000 at baseline and follow-up.<br><br>Most of the health promotion activities developed by the wellness committees were directed at improving diet (e.g., healthy snacks at meetings) or increasing physical activity (e.g., walking clubs), but others were more broadly focused, such as stress management or training in cardiopulmonary resuscitation (CPR) and first aid.<br><br>In the second year of the intervention, the research study sponsored an interschool competition and awarded cash prizes for high levels of participation in wellness activities. In addition, the study sponsored several programs for employees at all intervention schools, such as a healthy cooking class and a quarterly newsletter.

**Intervention delivery**						
**Who?**	NR	NR	School nurses (no details reported)	Workshops were delivered by wellness counsellors trained by Johnson and Johnson staff	Research staff (no details reported)	NR
**How?**	NR	Face-to-face; group-based training; followed up via telephone	Face-to-face; individual screening and counselling	Face-to-face; group-based workshops	Face-to-face; group-based activities	Face-to-face; group-based activities
**Where?**	Participating elementary schools	Participating elementary schools	Participating elementary schools	Participating elementary schools	Participating elementary schools	Participating middle schools
**When?**	NR	One single workshop	One single screening and counselling occasion	Weekly after-school workshops.<br><br>Exercise activities were generally held 2 to 3 times per week after school.	Ongoing activities for 2 years	One single nutrition training occasion for teachers (90 minutes)<br><br>Monthly peer support activities (30 minutes each)<br><br>Monthly meetings of the school nutrition groups
**Comparator condition**	1) HPS not aiming at dietary intervention (HP-ND)<br>2) Usual practice, i.e., non-health-promoting (NHP) schools	No intervention control group	No intervention control group	Comparison group schools received the Gimme-5 program, a health education curriculum designed to increase students' consumption of fruit and vegetables.	No intervention control group	No intervention control group

Note. This table includes excerpts from the published articles; the selection of items for which data were extracted was based to the TIDIER checklist; NR = not reported.
